# Effects of Legume–Grass Mixture Composition and Seeding Ratio on Plant Community Traits, Soil Physicochemical Properties, and Soil Fungal Diversity

**DOI:** 10.3390/jof12050353

**Published:** 2026-05-11

**Authors:** Jianyue Li, Jien Xi, Yuanwu Yang, Wei Wang, Chengti Xu, Jinling Ma, Xixi Yao, Haodong Liu

**Affiliations:** 1 College of Forestry and Grassland, Qinghai University, Xining 810016, China; jianyueli2026@163.com (J.L.); 13997162796@139.com (J.X.); yyuanwu@163.com (Y.Y.); 1624049718@qhu.edu.cn (J.M.); 2 Academy of Animal Husbandry and Veterinary Sciences, Qinghai University, Xining 810016, China; qibo780210@163.com (W.W.); xchti@163.com (C.X.); 3 College of Animal Husbandry and Veterinary Science, Gansu Polytechnic College of Animal Husbandry and Engineering, Wuwei 733006, China

**Keywords:** alpine grassland, legume–grass mixture, soil fungal community structure, plant community traits, soil physicochemical properties

## Abstract

Legume–grass mixtures are widely used to improve productivity and soil quality in alpine grasslands; however, their effects on soil fungal communities and the underlying mechanisms remain unclear. In this study, a two-factor field experiment (species richness (three, four, and five species) × legume–grass ratio (4:6, 3:7 and 2:8)) was conducted in an alpine artificial grassland on the Qinghai–Tibetan Plateau. Soil fungal communities were assessed using high-throughput sequencing combined with multivariate analyses. The results showed that *Ascomycota*, *Mortierellomycota*, and *Basidiomycota* were the dominant fungal phyla. Both species composition and seeding ratio significantly influenced fungal community structure and α-diversity primarily through indirect pathways mediated by plant community characteristics and soil properties. Plant height and soil total phosphorus (TP) were identified as key drivers of fungal α-diversity. Specifically, the four-species mixture (Z2) at a 3:7 legume–grass ratio resulted in relatively higher and more stable aboveground biomass and improved soil nutrient status, whereas increasing species richness to five species did not further enhance these benefits. Overall, our findings indicate that optimizing species composition and legume–grass ratio, rather than simply increasing species richness, is more effective for regulating soil fungal diversity and ecosystem function.

## 1. Introduction

Soil fungi are one of the key biological groups driving material cycling and energy flow in terrestrial ecosystems. They play fundamental roles in organic matter decomposition, nutrient mineralization, and the regulation of plant–soil feedback processes [1,2,3]. As primary decomposers in soil food webs, fungi are capable of degrading complex organic compounds and releasing essential nutrients such as carbon (C) and nitrogen (N) for plant uptake [4]. Previous studies have demonstrated that fungi can decompose recalcitrant substrates that are inaccessible to many other microorganisms [5]. In addition, fungal activity influences soil physical and chemical properties, contributes to nutrient cycling, enhances soil fertility, and plays a crucial role in maintaining soil quality and promoting plant growth [6].

In legume–grass mixed sowing systems, plant species composition and configuration play an important role in shaping the assembly of soil fungal communities by altering root exudate composition, litter input characteristics, and soil nutrient availability [7,8,9]. Compared with monoculture forage systems, legume–grass mixtures generally exhibit higher productivity, improved forage quality, enhanced soil fertility, and more efficient resource utilization [10]. Numerous studies have shown that multispecies mixtures can improve soil physicochemical properties, increase soil organic matter content, stimulate nitrogen cycling, and influence soil microbial diversity [11]. However, in contrast to bacterial communities, soil fungal communities—particularly in legume–grass systems under alpine conditions—remain relatively understudied, despite their dominant role in decomposition processes and nutrient turnover [12]. Emerging evidence suggests that fungi are especially sensitive to shifts in plant functional composition, yet their responses to coordinated changes in species richness and functional group proportion are still poorly understood [13].

Menyuan County in Qinghai Province, located on the northeastern margin of the Qinghai–Tibetan Plateau, is characterized by a typical alpine continental climate [14]. In this region, traditional monocropping practices often result in soil degradation and biodiversity loss, reduce soil microbial diversity and richness, and alter fungal community composition [15]. Therefore, adopting multispecies mixed sowing systems to improve land-use efficiency and ecosystem stability is of considerable ecological and agronomic importance. Most previous studies have focused primarily on comparisons between mixed and monoculture systems or on the effects of management practices (e.g., fertilization and mowing) on soil microbial communities [7,16,17]; few have explicitly disentangled the effects of species richness and legume–grass ratio. Unlike previous studies, this study employs a factorial experimental design that explicitly decouples species richness and legume–grass ratio, allowing their individual and interactive effects on soil fungal communities to be quantified under alpine and alkaline conditions. The selected richness levels (three to five species) and legume–grass ratios (4:6, 3:7, and 2:8) represent realistic gradients commonly used in alpine forage systems, reflecting variations in functional group dominance and nitrogen input from legumes [18,19]. More importantly, the mechanistic linkages among plant community traits, soil properties, and fungal community structure, as well as their relative contributions remain insufficiently resolved.

To address these gaps, we investigated how legume–grass mixture configurations regulate soil fungal community assembly under alpine conditions, focusing on the combined effects of species richness and legume–grass ratios. Specifically, this study addresses two key scientific questions: (1) to what extent species richness and mixture ratios govern fungal community assembly via environmental filtering and resource competition, and (2) which plant traits and soil properties serve as primary regulators of fungal diversity within the plant–soil–fungi system. Our findings aim to provide a theoretical basis for optimizing legume–grass mixtures and to inform sustainable management strategies in fragile alpine ecosystems.

## 2. Materials and Methods

### 2.1. Overview of the Study Area

The field experiment was conducted at the Forage Industry Technology Platform Experimental and Demonstration Base in Shagouliang Village, Beishan Township, Menyuan County, Haibei Prefecture, Qinghai Province, China (37°41′37″ N,102°02′07″ E). The experimental site covers an area of approximately 1.33 ha (20 mu) and is primarily used for fenced field trials and plot-based experimental research. The region experiences long sunshine duration (739.8–2264.8 h annually) and strong solar radiation (54.0–130.68 kcal cm^−2^ year^−1^), with pronounced diurnal temperature fluctuations. The mean annual air temperature is 0.8 °C, with a large diurnal temperature range (7.5–11.6 °C). The mean annual precipitation is approximately 520 mm, while the annual evaporation is about 1000 mm. The soil at the experimental site is alkaline, with a pH of 8.30. Initial soil physicochemical properties (0–20 cm depth) included a soil organic carbon (SOC) content of 33.51 g kg^−1^, total nitrogen (TN) of 1.59 g kg^−1^, and total phosphorus (TP) of 1.41 g kg^−1^.

### 2.2. Experimental Design

The field experiment was conducted from 15 April to 30 October 2024, covering the entire growing season under alpine continental climatic conditions. Soil and plant samples were collected on 1 October 2024 at the end of the growing season. The plant materials used in this study included forage pea (*Pisum sativum* L.) Qingjian No. 1 (provided by the Qinghai Academy of Animal Science and Veterinary Medicine), common vetch (*Vicia sativa* L.; provided by Qinghai Xinrui Agriculture and Animal Husbandry Technology Development Co., Ltd., Xining, China), milk vetch (*Astragalus adsurgens* Pall.) Ximu 333A (provided by the Qinghai Academy of Animal Science and Veterinary Medicine), forage oat (*Avena sativa* L.) Qingyin No. 1 (provided by the Qinghai Academy of Animal Science and Veterinary Medicine), and *triticale* (×*Triticosecale* Wittmack) Qingsimai No. 2 (provided by the Northwest Institute of Plateau Biology, Chinese Academy of Sciences). Among these species, *Pisum sativum*, *Vicia sativa*, and *Astragalus adsurgens* are legumes, whereas *Avena sativa* and *×Triticosecale* belong to grasses. This classification was used to define the legume-grass ratios in the experimental design. These forage species were selected because they are widely cultivated and well adapted to alpine conditions, with stable production performance. Legumes enhance soil nitrogen availability through biological nitrogen fixation, whereas grasses provide high biomass and efficient resource utilization. Their combination enables functional complementarity, thereby improving productivity and influencing soil fungal community structure.

A factorial field experiment with nested species composition was conducted to investigate the effects of legume–grass mixture configuration on plant–soil–fungal interactions. Species richness (Z) and legume–grass seeding ratio (B) were treated as the two main experimental factors. The species richness factor included three levels: Z1 (three-species mixture), Z2 (four-species mixture), and Z3 (five-species mixture). The seeding ratio factor also comprised three levels based on the legume-to-grass ratio: B1 (4:6), B2 (3:7), and B3 (2:8). The legume–grass ratios of 4:6, 3:7, and 2:8 were selected to represent commonly used seeding proportions in forage production systems. In addition, these ratios establish a gradient of decreasing legume proportion, enabling a systematic evaluation of how variations in legume contribution affect plant community characteristics, soil physicochemical properties, and soil fungal community structure [20]. Within each species richness level, three different species compositions were established as nested treatments to minimize species identity effects. Each treatment combination was replicated three times in a randomized block design, resulting in a total of 63 experimental plots. Each plot measured 3 m × 5 m (15 m^2^) and contained 10 planting rows with a row spacing of 30 cm, and the total experimental area covered approximately 0.33 ha (≈5 mu). Detailed information on species combinations, mixture ratios, and seeding rates are provided in Table 1. Aboveground biomass was harvested on 1 October 2024, to determine forage yield. No basal fertilizer, topdressing, or irrigation was applied during the experimental period, and soil fertility across the experimental site was relatively uniform. Routine field management practices, including manual weed removal, were carried out throughout the growing season.

### 2.3. Plant Community Survey and Soil Sample Collection

#### 2.3.1. Investigation of Plant Community

Within each plot, three 50 cm × 50 cm quadrats were randomly selected for vegetation assessment. Plant height was measured using a measuring tape, and aboveground biomass was determined concurrently. Community density was assessed using a direct counting method by recording the number of individual plants within each quadrat, with one measurement conducted per quadrat. All plants within each quadrat were clipped at ground level, leaving a stubble height of approximately 5 cm. The harvested samples were placed into paper bags and transported to the laboratory, where they were oven-dried at 65 °C for 72 h until constant weight was achieved.

#### 2.3.2. Collection of Soil Samples

Soil samples were collected on 1 October 2024 at the end of the growing season, coinciding with plant sampling. In the quadrats used for aboveground biomass determination, three soil cores were randomly collected using a soil auger (6 cm in diameter) from the 0–15 cm soil layer and subsequently combined into one composite sample per plot. The soil samples were transported to the laboratory and passed through a 2 mm sieve to remove stones and plant residues, and visible roots were carefully separated. The homogenized soil samples were then divided into three subsamples according to analytical requirements. The first subsample was air-dried at room temperature in the shade for the analysis of soil chemical properties, including soil pH, soil organic carbon (SOC), total nitrogen (TN), total phosphorus (TP), total potassium (TK), available nitrogen (AN), and available potassium (AK). The second subsample was stored at −20 °C for the determination of soil moisture content, which was measured using the gravimetric method by drying at 105 °C until constant weight was achieved, and was analyzed within one week after sampling. The third subsample was immediately stored at −80 °C for subsequent high-throughput sequencing analysis of the soil fungal community [21].

### 2.4. Determination of Soil Indicators

#### 2.4.1. Determination of Soil Physical and Chemical Properties

Soil physicochemical properties, including total nitrogen (TN), total phosphorus (TP), total potassium (TK), soil moisture content, soil organic carbon (SOC), PH, available nitrogen (AN), and available potassium (AK), were determined according to the standard procedures described in Soil Agrochemical Analysis (3rd Edition) [22].

#### 2.4.2. Soil Fungal DNA Extraction, PCR Amplification, and High-Throughput Sequencing

Soil fungal DNA extraction, PCR amplification, and high-throughput sequencing were conducted by Majorbio Bio-Pharm Technology Co., Ltd. (Shanghai, China). Total genomic DNA was extracted from soil samples using the E.Z.N.A.^®^ Soil DNA Kit (Omega Bio-tek, Norcross, GA, USA) according to the manufacturer’s instructions. The quality of the extracted DNA was assessed by 1% agarose gel electrophoresis, and DNA concentration and purity were determined using a NanoDrop 2000 spectrophotometer (Thermo Fisher Scientific, Waltham, MA, USA). Qualified DNA samples were stored at −80 °C until further analysis. The fungal internal transcribed spacer (ITS) region was amplified using the primer pair ITS1F (5′-CTTGGTCATTTAGAGGAAGTAA-3′) and ITS2R (5′-TCCTCCGCTTATTGATATGC-3′). PCR reactions were performed in a 20 μL mixture containing 4 μL of 5× FastPfu buffer, 2 μL of 2.5 mM dNTPs, 0.8 μL of each primer (5 μM), 0.4 μL of FastPfu DNA polymerase, 0.2 μL of bovine serum albumin (BSA), 10 ng of template DNA, and nuclease-free water to volume. All samples were amplified in triplicate. The PCR amplification program consisted of an initial denaturation at 95 °C for 3 min; 27 cycles of denaturation at 95 °C for 30 s, annealing at 60 °C for 30 s, and extension at 72 °C for 30 s; followed by a final extension at 72 °C for 10 min. PCR products were verified by 2% agarose gel electrophoresis, purified using magnetic beads, and quantified using a Qubit 4.0 fluorometer (Thermo Fisher Scientific, Waltham, MA, USA). Paired-end sequencing (2 × 250 bp) was performed on the Illumina MiSeq platform (Illumina, San Diego, CA, USA) by Majorbio Bio-Pharm Technology Co., Ltd. (Shanghai, China). Raw sequencing reads were quality-filtered using fastp (version 0.19.6) and merged using FLASH (version 1.2.11). Reads were truncated at positions where the average quality score fell below 20 within a 50 bp sliding window, and reads shorter than 50 bp or containing ambiguous bases were discarded. Paired-end reads were merged with a minimum overlap length of 10 bp and a maximum mismatch ratio of 0.2. The optimized sequences were clustered into operational taxonomic units (OTUs) at 97% similarity using UPARSE (version 7.1), and chimeric sequences were identified and removed. To reduce bias caused by sequencing depth, all samples were rarefied to an equal number of sequences prior to downstream analyses. Taxonomic assignment was performed using the RDP classifier (version 2.11) against the UNITE fungal ITS database (version 8.0) with a confidence threshold of 70%. Chloroplast and mitochondrial sequences were removed prior to analysis.

### 2.5. Data Analysis

Raw data were organized using Microsoft Excel 2019. Plant community characteristics, soil physicochemical properties, and soil fungal α-diversity indices were expressed as mean ± standard error (SE, *n* = 3). A two-way ANOVA was conducted with species richness (Z) and legume–grass ratio (B) as fixed factors, including their interaction. Species composition was treated as a nested factor within species richness to account for variation among different species combinations within each richness level. Significant differences among treatments were determined using the least significant difference (LSD) test at *p* < 0.05. Although LSD may increase the risk of Type I error under multiple comparisons, it was retained for consistency with previous ecological studies, and results were interpreted cautiously. For community composition analyses (e.g., Venn diagrams), fungal data were pooled across nested species compositions within each Z × B combination to represent overall treatment-level patterns. For statistical analyses, plot-level data were retained with species composition treated as a nested factor. High-throughput sequencing data of the fungal ITS region were processed using standard bioinformatic procedures. Rarefaction curves, community composition profiles, and relative abundance plots were generated. α-diversity indices, including Sobs, ACE, Chao1, and Shannon indices, were calculated. Functional prediction of soil fungal trophic modes and ecological guilds was conducted using FUNGuild. Only assignments with “probable” and “highly probable” confidence levels were retained for analysis. It should be noted that FUNGuild predictions are based on existing databases and should be considered as exploratory. Pearson correlation analysis and Mantel tests were performed in R software (version 4.2.0) to explore relationships among plant community characteristics, soil physicochemical properties, and fungal community attributes. Redundancy analysis (RDA) was carried out using Canoco 5.0 to quantify the explanatory power of plant and soil variables on fungal α-diversity and community structure at the phylum and genus levels. Structural equation modeling (SEM) was applied to assess the direct and indirect effects of grass species combinations, mixture ratios, and soil properties on the soil fungal community. In the SEM analysis, latent variables were defined as follows: plant community characteristics (plant height, density, aboveground biomass), soil properties (pH, SOC, TN, TP, TK, AN, AK), and fungal community attributes (α-diversity indices and dominant taxa). Model fit was evaluated using goodness-of-fit (GOF) and additional indices including SRMR. Non-significant paths were removed stepwise to obtain the final model. Figures were primarily generated using OriginPro 2024 and GraphPad Prism 9.5.0. Visualization of fungal community data was conducted on the Majorbio Cloud Platform (Majorbio Bio-Pharm Technology Co., Ltd., Shanghai, China).

## 3. Results

### 3.1. Effects of Legume–Grass Mixture Combinations and Ratios on Plant Community Characteristics

Significant differences in plant height, aboveground biomass, and plant density were observed among different legume–grass mixture combinations and ratios (Figure S1). Overall, mean plant height and aboveground biomass were highest under the Z2 treatment, followed by Z3 and Z1 (Z2 ≥ Z3 > Z1), whereas plant density showed the opposite trend, with the highest mean values observed under Z1. Aboveground biomass reached the highest values at the intermediate legume–grass ratio (3:7), particularly under Z2. Plant height increased with increasing grass proportion, whereas plant density decreased with increasing species richness. Within Z2, plant density was higher under the 4:6 ratio compared with other ratios (see Figure S1 for full details).

### 3.2. Effects of Legume–Grass Mixture Combinations and Ratios on Soil Physicochemical Properties

Significant differences were observed in TN, TP, TK, SOC, pH, and AK among treatments, whereas soil water content and available nitrogen showed no significant variation. Across mixture combinations, the Z2 treatment exhibited higher levels of TN, TP, TK, and SOC compared with Z1 and Z3. Intermediate legume–grass ratios (3:7 and 2:8) further increased nutrient levels under Z2. Soil pH was higher under Z3, with values reaching up to 9.16.

### 3.3. Effects of Legume–Grass Mixture Combinations and Ratios on Soil Fungal Community Structure and Diversity

#### 3.3.1. OTU Richness and Distribution

**Figure 1:**
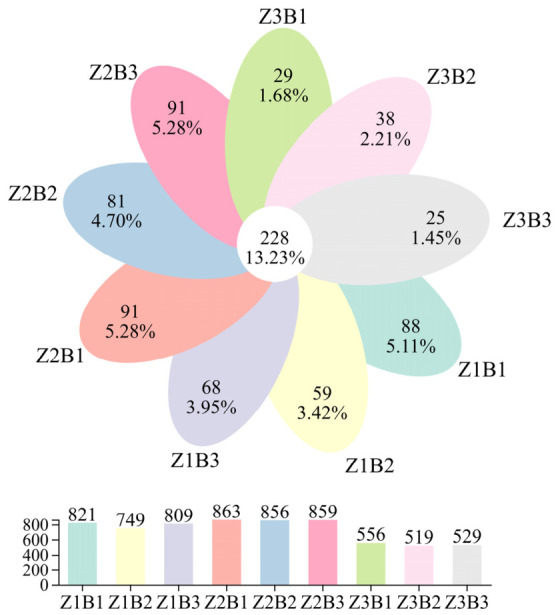
Venn analysis of fungal communities in soil samples. Venn diagram showing shared and unique OTUs among treatments. Z1, Z2, and Z3 represent three-, four-, and five-species mixtures, respectively. B1, B2, and B3 represent legume-to-grass ratios of 4:6, 3:7, and 2:8, respectively.

Venn analysis revealed clear differences in OTU distribution among treatments. The Z2B1 treatment exhibited the highest number of OTUs (863). In contrast, the Z3B1, Z3B2, and Z3B3 treatments showed the lowest OTU numbers, with 556, 519, and 529 OTUs, respectively. A total of 228 OTUs were shared among all nine treatments, accounting for 13.23% of the total detected OTUs (Figure 1).

#### 3.3.2. Changes in Soil Fungal Alpha Diversity

**Figure 2:**
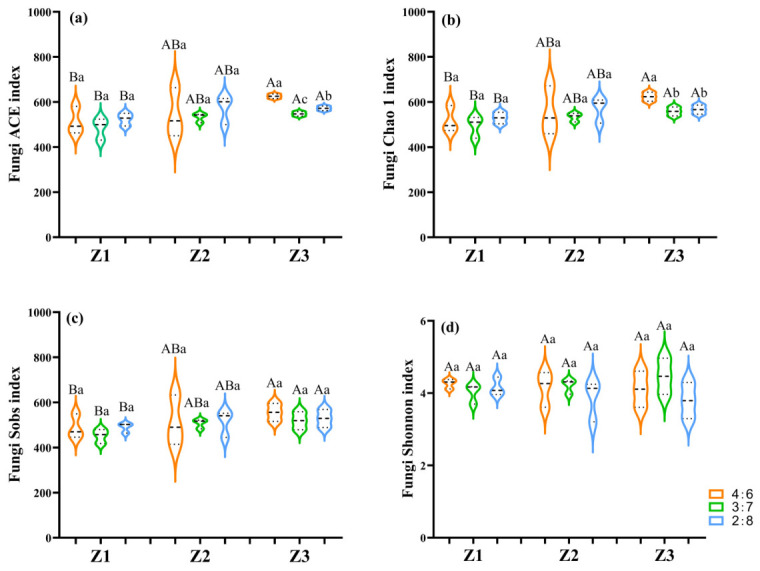
Differences in fungal alpha-diversity indices in soil samples under different legume–grass mixture combinations and ratios. (**a**) ACE index; (**b**) Chao1 index; (**c**) Sobs index; (**d**) Shannon index. Z1, Z2, and Z3 represent three-, four-, and five-species mixtures, respectively. Orange, green, and blue bars indicate legume-to-grass ratios of 4:6, 3:7, and 2:8, respectively. Different uppercase letters indicate significant differences among species combinations, whereas different lowercase letters indicate significant differences among mixture ratios within the same species combination (*p* < 0.05).

Grass species combinations and mixture ratios significantly regulated soil fungal alpha diversity. Under the Z3 treatment, both the ACE and Chao1 indices were significantly higher than those under Z1 (*p* < 0.05). Within Z3, the 4:6 ratio exhibited significantly higher ACE and Chao1 values than the 3:7 and 2:8 ratios (*p* < 0.05). The Sobs index under Z3 reached 534.67, which was significantly higher than that under Z1. The Sobs value under Z2 was 510.67 and did not differ significantly from those under Z1 and Z3 (Figure 2c). The Shannon index values under Z1, Z2, and Z3 were 4.14, 4.12, and 4.07, respectively. Within Z2, the highest Shannon index (4.20) was observed at the 3:7 ratio; however, differences among treatments were not statistically significant (Figure 2d).

#### 3.3.3. Changes in Soil Fungal Community Composition and Relative Abundance

**Figure 3:**
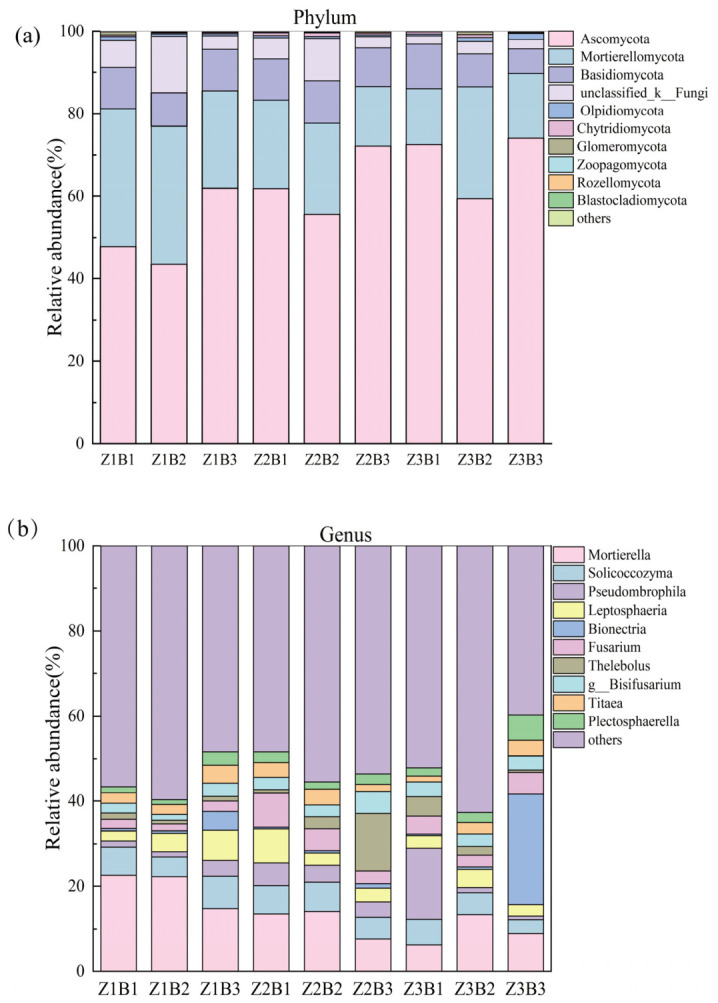
(**a**) Variation in the relative abundance of soil fungal communities at the phylum level. (**b**) Variation in the relative abundance of soil fungal communities at the genus level.

Significant variations were observed in the relative abundance of the top 10 fungal phyla in soil under different legume–grass mixture combinations and ratios (Figure 3a). *Ascomycota* (43.58–74.06%) was the dominant phylum across all treatments, followed by *Mortierellomycota* (13.55–33.40%) and *Basidiomycota* (6.02–10.77%). Significant differences in fungal phylum-level abundance were detected among treatments. The highest relative abundance of *Ascomycota* was observed under the Z3B3 treatment (74.06%). Under the Z3B3 treatment, the relative abundance of Mortierellomycota was 15.62%, which was higher than that under the Z2B3 (14.49%) and Z3B1 (13.55%) treatments. The relative abundance of Mortierellomycota under the Z1B1 and Z1B2 treatments was 33.39% and 33.35%, respectively, indicating minimal variation between the two treatments. At the genus level, significant differences were also observed in the relative abundance of the top 10 fungal genera among treatments (Figure 3b). Across the nine mixture treatments, the five most abundant genera were *Mortierella* (6.21–22.58%), *Solicoccozyma* (3.23–7.55%), *Pseudombrophila* (0.85–16.70%), *Leptosphaeria* (2.34–7.88%), and *Bionectria* (0.36–25.99%). Under the Z3B1 treatment, only *Mortierella* was detected among the dominant genera, with a relative abundance of 6.21%. The relative abundance of Fusarium under the Z2B1 and Z2B2 treatments was 7.95% and 5.09%, respectively, which was higher than that observed under most other treatments.

#### 3.3.4. Effects of Different Legume–Grass Mixture Combinations and Ratios on Soil Fungal Functional Guilds

**Figure 4:**
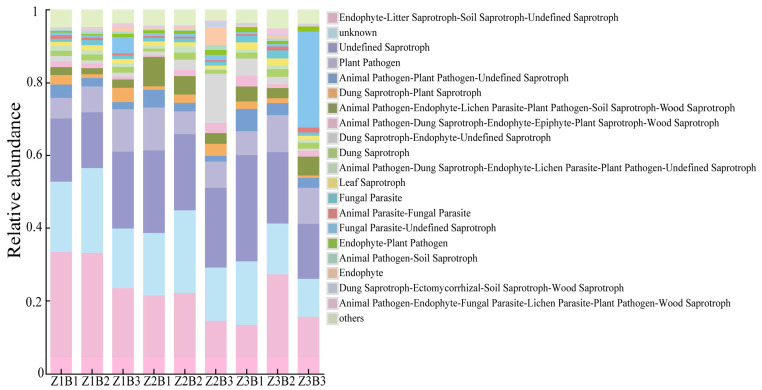
Functional classification of soil fungal communities based on FUNGuild analysis.

Based on FUNGuild functional prediction, a total of 20 fungal trophic modes were identified across the nine mixture treatments (Figure 4). It should be noted that a considerable proportion of sequences were classified as “Unknown” or “Undefined Saprotroph,” reflecting the limited resolution of current databases. In addition, FUNGuild assignments are based on probabilistic matching and should therefore be interpreted with caution. The five most abundant functional guilds were Endophyte–Litter Saprotroph–Soil Saprotroph–Undefined Saprotroph (13.35–33.38%), Unknown (10.32–23.24%), Undefined Saprotroph (15.13–29.13%), Plant Pathogen (5.60–11.84%), and Animal Pathogen–Plant Pathogen–Undefined Saprotroph (1.55–6.05%). Among treatments, Z1B1 and Z1B2 were dominated by Endophyte–Litter Saprotroph–Soil Saprotroph–Undefined Saprotroph, with relative abundances of 33.38% and 33.18%, respectively. The Z2B2 treatment was characterized by the highest proportion of Unknown (22.82%), which was significantly greater than that in the other treatments. The dominant guild under the Z1B3 and Z3B2 treatments were Undefined Saprotroph, with relative abundances of 16.43% and 14.00%, respectively. In contrast, under the Z3B3 treatment, the dominant functional guild was Fungal Parasite–Undefined Saprotroph, with a relative abundance of 26.30%. Notably, the lowest relative abundance of Plant Pathogen was observed under the Z1B1 treatment (5.60%). The observed shifts in fungal functional guilds may be associated with changes in plant community characteristics and soil properties. For example, higher proportions of saprotrophic groups under Z2 treatments may be linked to increased aboveground biomass and enhanced organic matter inputs, while variations in phosphorus availability (TP) may further influence the distribution of functionally distinct fungal groups.

#### 3.3.5. Clustered Heatmap Analysis of Soil Fungal Communities

**Figure 5:**
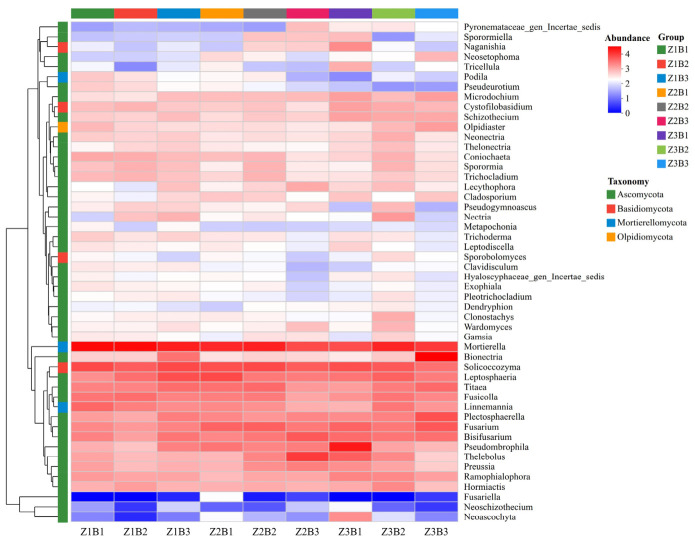
Clustered heatmap of soil fungal communities at the genus level.

Phylogenetic heatmap and hierarchical clustering analyses at the genus level revealed that the nine mixture treatments exerted pronounced regulatory effects on soil fungal community composition (Figure 5). The top 50 most abundant fungal genera exhibited clear differences among treatments, but also showed consistent ecological patterns. Across all treatments, *Mortierella* remained the dominant genus and served as a key hub taxon, indicating its central role in soil nutrient cycling under alpine conditions. In addition, several genera, such as *Solicoccozyma* and *Leptosphaeria*, were widely distributed across treatments, suggesting a broad ecological tolerance to variations in species composition and environmental conditions. Differences among treatments were mainly reflected in the relative enrichment of specific genera under different mixture configurations. For example, genera such as *Fusarium* and *Titaea* tended to show higher relative abundance under moderate species richness (Z2), whereas some taxa (e.g., *Pseudombrophila* and *Bionectria*) were more frequently associated with higher richness levels (Z3). These patterns suggest that plant species composition and legume-grass ratios may influence fungal community assembly by modifying ecological niches and resource availability. Overall, genus-level community structure was jointly regulated by plant diversity and soil environmental conditions, rather than being determined by individual treatments alone.

#### 3.3.6. Coupling Relationships Among Plant Community Characteristics, Soil Physicochemical Properties, and Soil Fungal Community Structure

**Figure 6:**
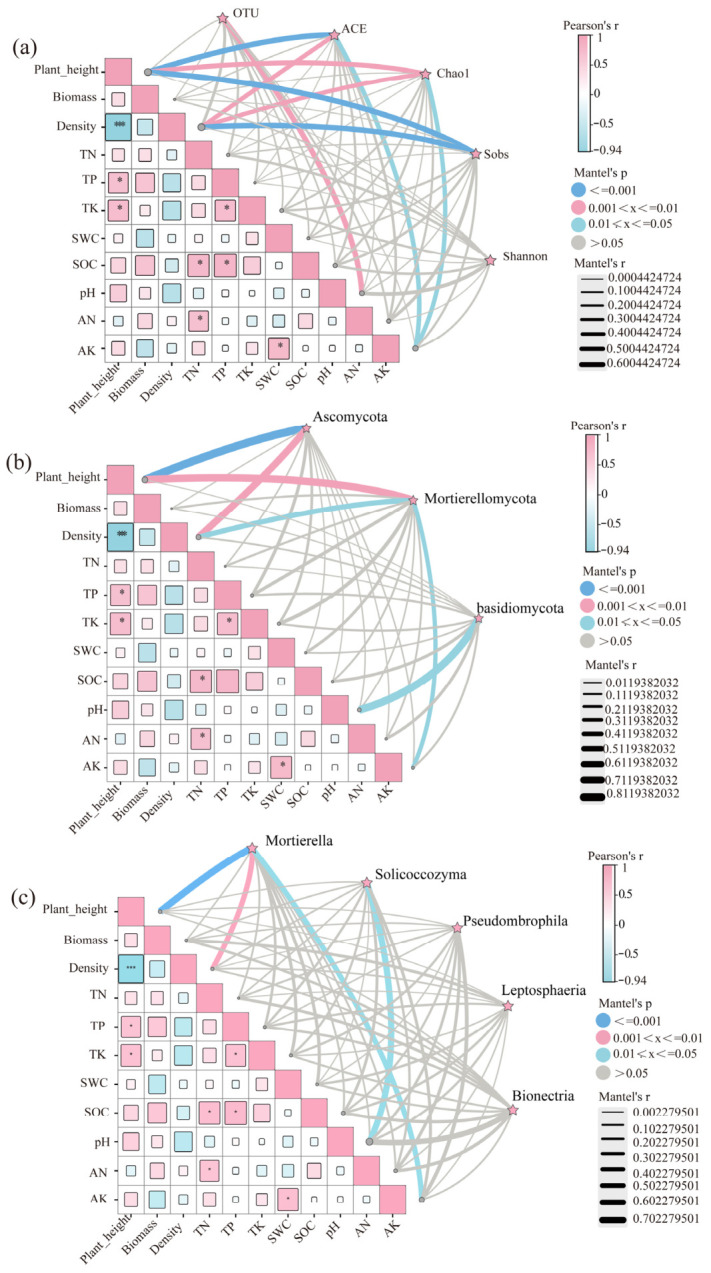
Correlation analysis among plant community characteristics, soil physicochemical properties, and soil fungal communities. (**a**) Alpha diversity; (**b**) fungal community at the phylum level; (**c**) fungal community at the genus level. Squares represent Pearson’s correlation coefficients between variables, with color indicating the strength and direction of the correlation (red: positive; blue: negative). The size of the squares reflects the magnitude of the correlation. Lines represent Mantel correlations between environmental factors and fungal communities. Line thickness indicates the strength of Mantel’s r, and color represents significance levels (blue: *p* ≤ 0.001; pink: 0.001 < *p* ≤ 0.01; light blue: 0.01 < *p* ≤ 0.05; grey: *p* > 0.05). Asterisks indicate statistical significance of Pearson’s correlations: * *p* < 0.05; *** *p* < 0.001.

Mantel analysis revealed significant correlations among plant community characteristics, soil physicochemical properties, and soil fungal community attributes (Figure 6). At the plant–soil level, plant height was significantly positively correlated with TP and TK (*p* < 0.05), while TN showed significant positive correlations with SOC and AN (*p* < 0.05). In addition, TP was positively correlated with SOC and TC, and SWC was positively correlated with AK (*p* < 0.05). In contrast, plant height was extremely significantly negatively correlated with plant density (*p* < 0.001), indicating a trade-off between plant size and density. To improve interpretability, we focused on the most robust relationships between environmental variables and fungal community attributes. At the α-diversity level (Figure 6a), fungal richness indices (ACE, Chao1, and Sobs) showed strong negative correlations with plant height and plant density, with particularly significant negative relationships observed for ACE and Sobs (*p* < 0.001). Both ACE and Chao1 indices were also significantly correlated with AK (*p* < 0.05), while the number of OTUs was significantly associated with available nitrogen (AN) (*p* < 0.01). In contrast, the Shannon index showed no significant correlations with plant or soil variables. At the phylum level (Figure 6b), Ascomycota and Mortierellomycota exhibited strong associations with plant community characteristics. Ascomycota was extremely significantly negatively correlated with plant height (*p* < 0.001) and significantly associated with plant density (*p* < 0.01), while Mortierellomycota was extremely significantly correlated with plant height (*p* < 0.01) and also showed significant correlations with plant density and AK (*p* < 0.05). In addition, Basidiomycota showed a significant correlation with soil pH (*p* < 0.05).

At the genus level (Figure 6c), *Mortierella* was significantly correlated with plant density (*p* < 0.01) and AK (*p* < 0.05), and exhibited an extremely significant negative correlation with plant height (*p* < 0.001). *Solicoccozyma* showed a significant correlation with soil pH (*p* < 0.05), whereas *Pseudombrophila*, *Leptosphaeria*, and *Bionectria* showed no significant associations with plant or soil variables. Overall, these results highlight that plant structural traits, particularly plant height, play a key role in shaping fungal community diversity and composition, suggesting that increased plant dominance may constrain fungal diversity through changes in resource availability and niche conditions.

**Figure 7:**
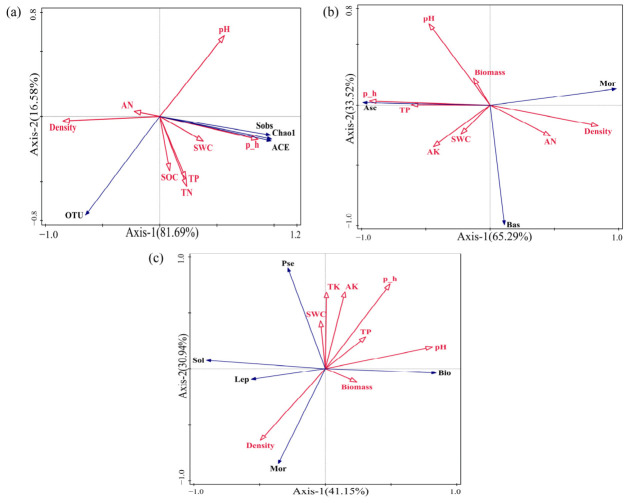
Redundancy analysis (RDA) of plant community characteristics, soil physicochemical properties, and soil fungal community structure. Red arrows represent plant community characteristics and soil environmental variables, whereas blue arrows represent fungal community attributes. (**a**) Alpha diversity; (**b**) fungal community at the phylum level; (**c**) fungal community at the genus level. Abbreviations: p_h, plant height; Density, plant density; TN, total nitrogen; TP, total phosphorus; TK, total potassium; SWC, soil water content; SOC, soil organic carbon; AN, available nitrogen; AK, available potassium.

Redundancy analysis (RDA) revealed that soil fungal α-diversity was influenced by soil physicochemical properties and plant community characteristics (Figure 7a). Soil pH and total phosphorus (TP) were identified as important environmental factors shaping fungal alpha diversity. The Sobs, Chao1, and ACE indices were negatively associated with plant density and available nitrogen (AN), but positively correlated with other environmental variables. At the phylum level, soil pH showed a marginally significant effect on fungal community structure (Figure 7b, *p* = 0.052), suggesting a potential but relatively weak influence. Basidiomycota showed negative correlations with TP, soil pH, and biomass, but positive associations with other environmental factors. At the genus level, soil pH remained an important factor influencing fungal community composition (Figure 7c). *Bionectria* was negatively correlated with plant density and soil water content (SWC), while showing positive correlations with other environmental variables.

#### 3.3.7. Potential Driving Factors of Soil Fungal Community Diversity

**Figure 8:**
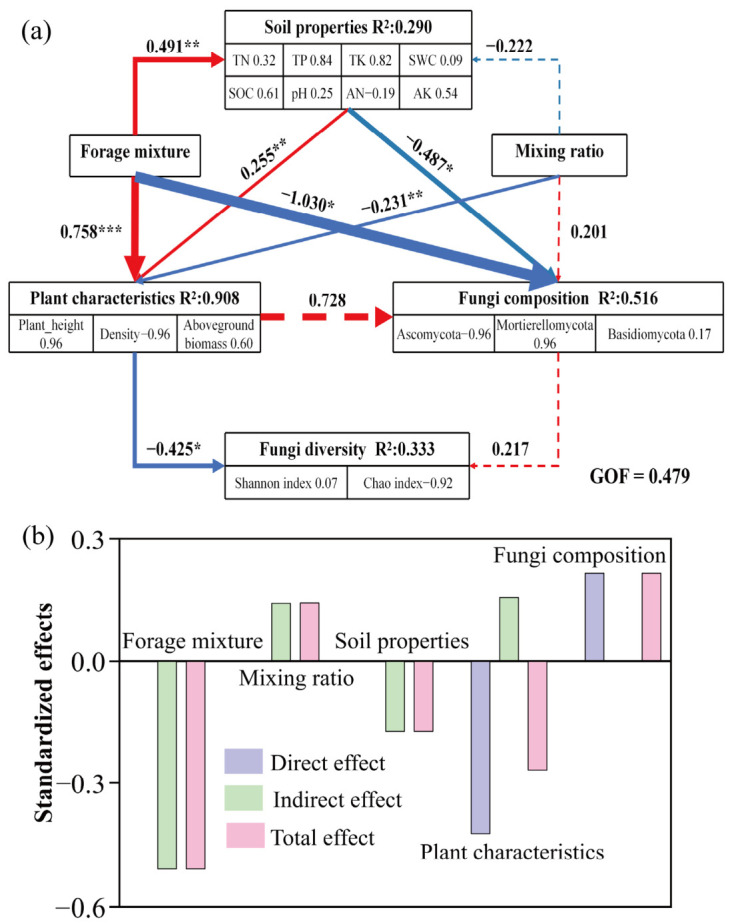
Structural equation model (SEM) based on PLS-PM. (**a**) Structural equation model illustrating the direct and indirect effects of grass species combination, mixture ratio, plant community characteristics, and soil physicochemical properties on fungal diversity. Line thickness represents the strength of the relationships (thicker lines indicate stronger correlations). Red and blue arrows indicate positive and negative relationships, respectively. Numbers adjacent to the arrows represent standardized path coefficients. The proportion of explained variance (R^2^) is shown next to each response variable in the model. (**b**) Bar plots showing the direct, indirect, and total effects of grass species combination, mixture ratio, plant community characteristics, soil physicochemical properties, and fungal community composition on fungal alpha diversity. Asterisks indicate statistical significance: * *p* < 0.05; ** *p* < 0.01; *** *p* < 0.001. Dashed lines indicate non-significant relationships (*p* > 0.05).

Structural equation modeling (SEM) analysis revealed that grass species combination significantly influenced plant community characteristics and soil physicochemical properties (Figure 8a), while soil physicochemical properties also had a positive effect on plant community characteristics. In contrast, soil properties and species combination were negatively associated with fungal community composition. Plant community characteristics and mixture ratio were negatively related to fungal α-diversity, whereas fungal community composition showed a positive but non-significant association. In this study, a “negative effect” in the SEM indicates an inverse relationship between variables rather than a detrimental ecological impact. For example, the negative association between plant community characteristics and fungal alpha diversity may reflect increased plant dominance and reduced niche heterogeneity, which can limit fungal diversity.

Further analysis indicated that fungal α-diversity was shaped by both direct and indirect pathways. Grass species combination mainly influenced fungal diversity indirectly via plant community characteristics and soil physicochemical properties, whereas mixture ratio showed an overall positive contribution. Fungal community composition exerted the strongest direct effect on α-diversity, highlighting its key role in shaping fungal diversity patterns (Figure 8b). It should be noted that the SEM results are based on cross-sectional data from a single growing season and therefore do not establish causality. In addition, potential collinearity among variables and the relatively limited sample size may increase the risk of model overfitting, which should be considered when interpreting the model results.

## 4. Discussion

### 4.1. Effects of Different Legume–Grass Mixture Combinations and Ratios on Artificial Grassland Community Characteristics

The present study demonstrated that different legume–grass mixture combinations and ratios significantly affected plant height, aboveground biomass, and plant density (Figure S1). Notably, the four-species mixture (Z2) achieved relatively higher and more stable biomass under appropriate mixture ratios (Figure S1b), suggesting that “moderate diversity” may outperform “extremely high diversity” in the construction of artificial grasslands in alpine regions. This finding is consistent with the results reported by Foster et al. [23]. The superior performance of the Z2 treatment may be attributed to an optimal balance between species complementarity and competition, allowing more efficient utilization of resources such as light, water, and nutrients. In legume–grass mixtures, complementary interactions among species in both horizontal and vertical space can enhance coordinated resource use, thereby promoting plant height. Accordingly, plant height under Z2 (four-species mixture) and Z3 (five-species mixture) was significantly greater than under Z1 (three-species mixture) (Figure S1a), likely due to earlier canopy development and more efficient light interception in species-rich communities [24]. In contrast, although the Z1 treatment exhibited higher plant density (Figure S1c), it did not produce the highest biomass, suggesting that excessive density intensifies competition for light, water, and nutrients, thereby restricting individual plant growth. This may lead to increased size inequality and competitive suppression, ultimately reducing overall productivity [25]. High-density conditions often reduce individual mature size, and total community biomass may exhibit an optimal density or diminishing returns pattern in response to increasing density [26]. Regarding mixture ratios, the 3:7 legume-to-grass ratio under the Z2 treatment resulted in the highest biomass (Figure S1b), which aligns with the findings of Bi et al. [27]. This pattern may be attributed to a balance between nitrogen input from legumes and biomass production from grasses, highlighting the importance of optimizing functional group proportions in alpine forage systems [28,29].

### 4.2. Effects of Different Legume–Grass Mixture Combinations and Ratios on Soil Physicochemical Properties

Soil nutrients are essential for promoting plant growth and represent key indicators of grassland ecosystem restoration [30]. Plant species composition can, in turn, regulate soil nutrient dynamics through litter input, root turnover, and rhizosphere interactions [31,32,33]. Therefore, different mixture combinations and ratios can indirectly influence soil physicochemical properties by regulating plant growth characteristics. The present study showed that legume–grass mixture combinations and ratios significantly affected total nitrogen (TN), total phosphorus (TP), total potassium (TK), soil organic carbon (SOC), and pH (Figure S2d–h), whereas their effects on available nitrogen (AN), available potassium (AK), and soil water content (SWC) were relatively limited. These findings are consistent with those of Chen et al. [6] and Wu et al. [34], suggesting that in alpine regions, soil available nutrients are more strongly regulated by meteorological factors such as precipitation and leaching, and respond less sensitively to short-term changes in planting patterns [35]. Among treatments, the four-species mixture (Z2) exhibited clear advantages in total nutrient accumulation, particularly under the 3:7 legume-to-grass ratio, where TN and SOC were significantly increased (Figure S2d,e), consistent with Bi et al. [27]. This may be attributed to enhanced carbon and nitrogen inputs derived from higher biomass production and improved litter quality. Legumes contribute nitrogen through biological fixation, while grasses provide greater biomass inputs, and their combination promotes both carbon accumulation and nitrogen enrichment in soil [36]. Moreover, TP and TK contents under Z2 were generally higher than those under Z1 and Z3 (Figure S2f,h), suggesting that moderate species diversity may enhance the activation and accumulation of soil mineral nutrients. The observed effects may be related to niche differentiation of plant root systems in both vertical and horizontal space, which reduces interspecific competition for soil resources [37]. Although the five-species mixture (Z3) increased species richness, soil nutrient levels did not continue to rise, possibly due to increased functional redundancy or intensified rhizosphere competition [38]. These findings suggest that increasing plant species richness does not necessarily enhance soil nutrient accumulation [39]. No significant effects of mixture combinations or ratios on available nitrogen (AN) were detected (Figure S2g). AN varied within a relatively narrow range (approximately 10% across treatments), and although slightly higher values were observed under the Z2 treatment, the differences were not statistically significant. In contrast, soil pH remained relatively stable across treatments; however, the overall pH values were relatively high (up to 9.16), indicating strongly alkaline conditions. Such alkaline environments may act as important environmental filters on soil fungal communities, potentially constraining the growth and activity of pH-sensitive taxa, particularly some Basidiomycota and symbiotic fungi that are typically associated with neutral to slightly acidic soils. In contrast, Ascomycota are generally more tolerant of alkaline and nutrient-variable environments, which may partly explain their dominance across treatments in this study [40]. Previous studies have shown that soil pH is a critical factor influencing fungal community structure and diversity [40], and its relative stability in this study may partly explain the moderate variation in fungal community composition among treatments [41]. It should be noted that no fertilizer was applied during the experiment, which may have resulted in nutrient limitations, particularly phosphorus, potentially influencing the observed responses of soil fungal communities.

### 4.3. Effects of Different Legume–Grass Mixture Combinations and Ratios on Soil Fungal Community Structure and Function

The number of fungal OTUs and α-diversity indices are important indicators reflecting soil ecosystem function and microbial community structure [41]. Different forage species combinations and legume–grass ratios can regulate soil fungal community structure and function by altering root exudate composition, litter input characteristics, and soil physicochemical conditions [42]. In this study, fungal OTU richness and diversity varied significantly among treatments (Figure 1 and Figure 6a), indicating that plant diversity and mixture patterns play an important role in shaping fungal community assembly in alpine grasslands. The four-species mixture (Z2) exhibited relatively higher and more stable OTU numbers across most treatments (Figure 1), suggesting that moderate species diversity may promote a more stable fungal community structure. Although the five-species mixture (Z3) significantly increased richness-related indices (ACE and Chao1) (Figure 6a), community structural stability and functional balance were not simultaneously enhanced. This suggests that, in alpine artificial grasslands, moderate species diversity may be more favorable for optimizing fungal community assembly [19,43]. At the α-diversity level, ACE and Chao1 indices under Z3 (five-species mixture) were significantly higher than those under Z1 (three-species mixture) (Figure 6a), indicating that increased plant species richness expands the potential fungal species pool. However, Shannon diversity did not differ significantly among mixture treatments, suggesting that higher richness did not necessarily enhance community evenness. This pattern may be attributed to intensified interspecific competition among fungal taxa under high-diversity conditions, leading to dominance by certain taxa [44]. In contrast, the Z2 treatment showed a more balanced pattern in OTU number and Shannon index, particularly under the 3:7 legume–grass ratio, where Shannon diversity reached its maximum (Figure 6a), indicating improved community stability.

At the phylum level, *Ascomycota*, *Mortierellomycota*, and *Basidiomycota* were dominant across all mixture treatments (Figure 3a), consistent with previous findings from alpine grasslands on the Qinghai–Tibetan Plateau and other cold-region ecosystems [45]. Soil physicochemical conditions, particularly soil pH and phosphorus availability (TP), are key factors influencing fungal community composition [46,47]. In this study, the relatively high soil pH (alkaline conditions) may favor the dominance of Ascomycota, which are generally more tolerant of alkaline and nutrient-variable environments, whereas Basidiomycota are often associated with more stable, organic matter-rich environments and may be less competitive under such conditions [48,49]. In addition, increased TP may promote the proliferation of fast-growing saprotrophic fungi, especially within Ascomycota, by enhancing nutrient availability for microbial metabolism and enzymatic activity. The ITS1F/ITS2 primer pair may bias against certain fungal taxa, particularly Basidiomycota, leading to their underrepresentation. This should be considered when interpreting fungal community composition.

*Ascomycota* was most abundant in the Z3B3 treatment (Figure 3a), possibly due to the higher proportion of grasses and greater litter input under this configuration. In contrast, Mortierellomycota showed the highest relative abundance in Z1B1 (Figure 3a), suggesting that lower species richness combined with a higher legume proportion may favor fungal groups associated with plant growth promotion and phosphorus cycling [50]. At the genus level, Mortierella dominated across all treatments and acted as a key functional taxon (Figure 3b). Its relative abundance was significantly correlated with plant density and available potassium (Figure 6c), indicating that it may contribute to mineral nutrient transformation and plant nutrient acquisition in alpine artificial grasslands [21]. Several other dominant genera may also play important ecological roles. For example, Pseudombrophila is associated with organic matter decomposition and may contribute to nutrient cycling. Bionectria includes taxa with saprotrophic and potential biocontrol functions, while Solicoccozyma is commonly found in soil and plant-associated environments and may contribute to stress tolerance and nutrient turnover. These functional characteristics suggest that shifts in genus-level composition reflect changes in decomposition processes and plant–microbe interactions [51].

Notably, distinct enrichment patterns of dominant genera were observed among mixture treatments. For example, *Fusarium* showed significantly higher relative abundance in Z2B1 and Z2B2 (Figure 3b), suggesting that moderate species richness and suitable legume–grass ratios may create ecological niches that facilitate the proliferation of certain opportunistic or potentially pathogenic fungi [52]. The increased relative abundance of Fusarium under certain Z2 treatments may be associated with changes in plant community structure and root exudate composition under moderate species richness. As some Fusarium species are known plant pathogens, this pattern may indicate a potential trade-off between enhanced productivity and increased disease risk, which warrants further investigation. It should be noted that the use of ITS1F/ITS2 primers may introduce amplification bias against certain fungal groups, particularly Basidiomycota, which may lead to their underrepresentation in sequencing results and should be considered when interpreting the findings.

FUNGuild-based functional prediction further revealed that mixture combinations and ratios significantly reshaped fungal functional structure (Figure 4). Compared with Z1, Z2 and Z3 treatments exhibited higher proportions of Undefined Saprotroph and composite saprotrophic functional groups, whereas the relative abundance of Plant Pathogen guilds decreased in some treatments. These results indicate that appropriate mixture configurations may promote the establishment of functionally beneficial fungal communities dominated by nutrient cycling and organic matter decomposition processes [53]. It should be noted that FUNGuild-based assignments are based on probabilistic matching and should therefore be interpreted with caution.

### 4.4. Correlations Among Plant Community Characteristics, Soil Physicochemical Properties, and Soil Fungal Diversity

The Mantel test revealed significant coupling relationships between plant community characteristics and soil physicochemical properties in different legume–grass mixture systems (Figure 6). Specifically, plant height was significantly positively correlated with soil total phosphorus (TP) and total potassium (TK), but showed a highly significant negative correlation with plant density. Total nitrogen (TN) was significantly correlated with soil organic carbon (SOC) and available nitrogen (AN), TP was significantly correlated with SOC, and soil water content (SWC) was significantly correlated with available potassium (AK) (Figure 6). These results indicate strong synergistic interactions among carbon, nitrogen, and mineral nutrient cycling processes in mixed-sowing systems [54,55,56]. Further analysis demonstrated significant associations between fungal α-diversity, phylum- and genus-level relative abundances, and plant–soil variables (Figure 6a–c). Specifically, ACE, Chao1, Sobs indices, as well as the relative abundances of *Ascomycota* and *Mortierella*, were significantly or highly significantly negatively correlated with plant height (Figure 6a–c). This pattern differs from the findings of Guo et al. [21], and may be explained by the negative relationship observed in this study between plant height and density. Taller plant communities were generally associated with lower density, potentially resulting in a more homogeneous aboveground structure and soil environment, thereby reducing fungal richness and the abundance of certain dominant taxa [36]. This negative relationship may reflect ecological trade-offs, whereby increased plant dominance reduces niche heterogeneity and limits the diversity of available substrates for soil microorganisms.

Redundancy analysis (RDA) further indicated that plant height (p_h) and soil TP were key environmental drivers of fungal α-diversity (Figure 7a), consistent with the results reported by Tomazelli and Graça et al. [57,58]. Soil pH and nutrient availability—particularly phosphorus—have been widely reported as key factors regulating fungal community structure and function, with pH often acting as a dominant driver [59]. These results suggest that plant community structure and soil nutrient status jointly regulate fungal diversity through coordinated aboveground–belowground interactions. In addition, plant height was the dominant factor shaping fungal community structure at the phylum level, whereas soil pH played a key role at the genus level (Figure 7b,c), consistent with the observations of Vylkova et al. [60]. Together, these results indicate a tight coupling between aboveground plant community structure and belowground chemical environments, which collectively determine fungal community assembly patterns [61].

Structural equation modeling (SEM) further demonstrated that forage species combinations and legume–grass ratios jointly influenced soil fungal α-diversity through both direct and indirect pathways mediated by plant community characteristics and soil physicochemical properties (Figure 8). This finding suggests that forage species configuration serves as a primary upstream driver of plant–soil system dynamics, consistent with previous studies indicating that plant species composition regulates belowground microbial processes by modifying community structure and soil nutrient conditions [62,63]. In the present study, both soil physicochemical properties and forage species combinations exerted significant direct effects on fungal community composition, indicating that these factors play important roles in shaping community structure under alpine conditions, where soil nutrient status and chemical constraints may act as environmental filters, limiting the coexistence of certain fungal taxa and thereby reshaping community structure. However, it should be noted that SEM results in this study are based on cross-sectional observational data and therefore cannot establish definitive causal relationships. Instead, the identified pathways represent statistical associations that are consistent with hypothesized causal mechanisms. Consequently, these relationships should be interpreted with caution.

Although fungal community composition showed a positive effect on α-diversity, this pathway was not statistically significant, indicating that changes in fungal diversity in mixed grasslands cannot be explained solely by shifts in dominant phyla or genera. Instead, fungal α-diversity appears to be regulated by the integrated effects of plant community structure and soil environmental conditions [64]. Overall, different legume–grass mixture configurations regulate fungal diversity through multiple coupled pathways. These findings highlight that in alpine artificial grassland restoration, simply increasing species richness or adjusting legume proportions may not linearly enhance soil fungal diversity. Instead, optimizing community structure and avoiding excessive dominance may be the more effective strategies for maintaining belowground microbial diversity and ecosystem stability [8,65]. It should be noted that this study did not consider interannual or seasonal dynamics of fungal communities. Long-term monitoring combined with multi-season sampling is therefore necessary to further evaluate the stability of plant–soil–fungus interactions under different mixture configurations.

## 5. Conclusions

This study demonstrated that in alpine regions of the Qinghai–Tibetan Plateau, a four-species forage mixture with a 3:7 legume-to-grass ratio was more effective in coordinating plant growth, soil nutrient status, and soil fungal community structure. This mixed-sowing configuration maintained relatively high aboveground biomass while promoting a fungal community dominated by Ascomycota and Mortierellomycota. Redundancy analysis and structural equation modeling further revealed that plant height and soil total phosphorus (TP) were key drivers of variation in fungal α-diversity. From a broader ecological perspective, our findings indicate that under alpine and strongly alkaline conditions, plant structural traits and soil nutrient availability jointly regulate fungal community diversity through both direct and indirect pathways. In particular, plant–soil interactions mediate the effects of species composition and mixture ratio on fungal community assembly, highlighting the importance of environmental filtering and resource-driven processes in shaping fungal diversity.

Overall, our results emphasize that optimizing forage species composition and legume–grass ratios, rather than simply increasing species richness, is a more effective strategy for achieving coordinated improvement in aboveground productivity and belowground microbial processes in alpine grasslands. It should be noted that this study was conducted at a single site and within one growing season. Therefore, the generality of these findings should be interpreted with caution, and further studies across multiple sites and longer temporal scales are needed to validate these patterns.

## Figures and Tables

**Table 1:** Species composition of legume–grass mixtures expressed as common names and percentages of total seed weight.

Group	Mixture	Species Composition (% of Total Seed Weight)
Z1	3-species mixture-1	Forage pea (40%), Oat (40%), Triticale (20%)
		Forage pea (30%), Oat (50%), Triticale (20%)
		Forage pea (20%), Oat (60%), Triticale (20%)
	3-species mixture-2	Common vetch (40%), Oat (40%), Triticale (20%)
		Common vetch (30%), Oat (50%), Triticale (20%)
		Common vetch (20%), Oat (60%), Triticale (20%)
	3-species mixture-3	Milk vetch (40%), Oat (40%), Triticale (20%)
		Milk vetch (30%), Oat (50%), Triticale (20%)
		Milk vetch (20%), Oat (60%), Triticale (20%)
Z2	4-species mixture-1	Forage pea (20%), Common vetch (20%), Oat (40%), Triticale (20%)
		Forage pea (15%), Common vetch (15%), Oat (50%), Triticale (20%)
		Forage pea (10%), Common vetch (10%), Oat (60%), Triticale (20%)
	4-species mixture-2	Common vetch (20%), Milk vetch (20%), Oat (40%), Triticale (20%)
		Common vetch (15%), Milk vetch (15%), Oat (50%), Triticale (20%)
		Common vetch (10%), Milk vetch (10%), Oat (60%), Triticale (20%)
	4-species mixture-3	Forage pea (20%), Milk vetch (20%), Oat (40%), Triticale (20%)
		Forage pea (15%), Milk vetch (15%), Oat (50%), Triticale (20%)
		Forage pea (10%), Milk vetch (10%), Oat (60%), Triticale (20%)
Z3	5-species mixture-1	Forage pea (10%), Common vetch (25%), Milk vetch (5%), oat (40%), Triticale (20%)
	5-species mixture-2	Forage pea (10%), Common vetch (15%), Milk vetch (5%), oat (50%), Triticale (20%)
	5-species mixture-3	Forage pea (10%), Common vetch (5%), Milk vetch (5%), oat (60%), Triticale (20%)

Note: All treatments were sown at a total seed rate of 90 kg ha^−1^. Percentages indicate the proportion of each species based on total seed weight. Abbreviations: Z1, three-species mixtures; Z2, four-species mixtures; Z3, five-species mixtures.

## Data Availability

The original contributions presented in this study are included in the article; further inquiries can be directed to the corresponding author.

## Supplementary Materials

The following supporting information can be downloaded at: https://www.mdpi.com/article/10.3390/jof12050353/s1, Figure S1. Changes in plant community characteristics under different legume–grass mixture combinations and ratios; Figure S2. Changes in soil physicochemical properties under different legume–grass mixture combinations and seeding ratios; Figure S3. Chord diagram showing the relationships between fungal phyla and different treatments; Figure S4. Chord diagram showing the relationships between dominant fungal genera and different treatments.
