# Supplementary material for: Effects of Legume–Grass Mixture Composition and Seeding Ratio on Plant Community Traits, Soil Physicochemical Properties, and Soil Fungal Diversity
Source: J Fungi (Basel). 2026 May 11;12(5):353. doi: 10.3390/jof12050353 (PMC13208266; doi:10.3390/jof12050353)
Supplement: Supplementary file 1 [file jof-12-00353-s001.zip › jof-4246271-supplementary.pdf]

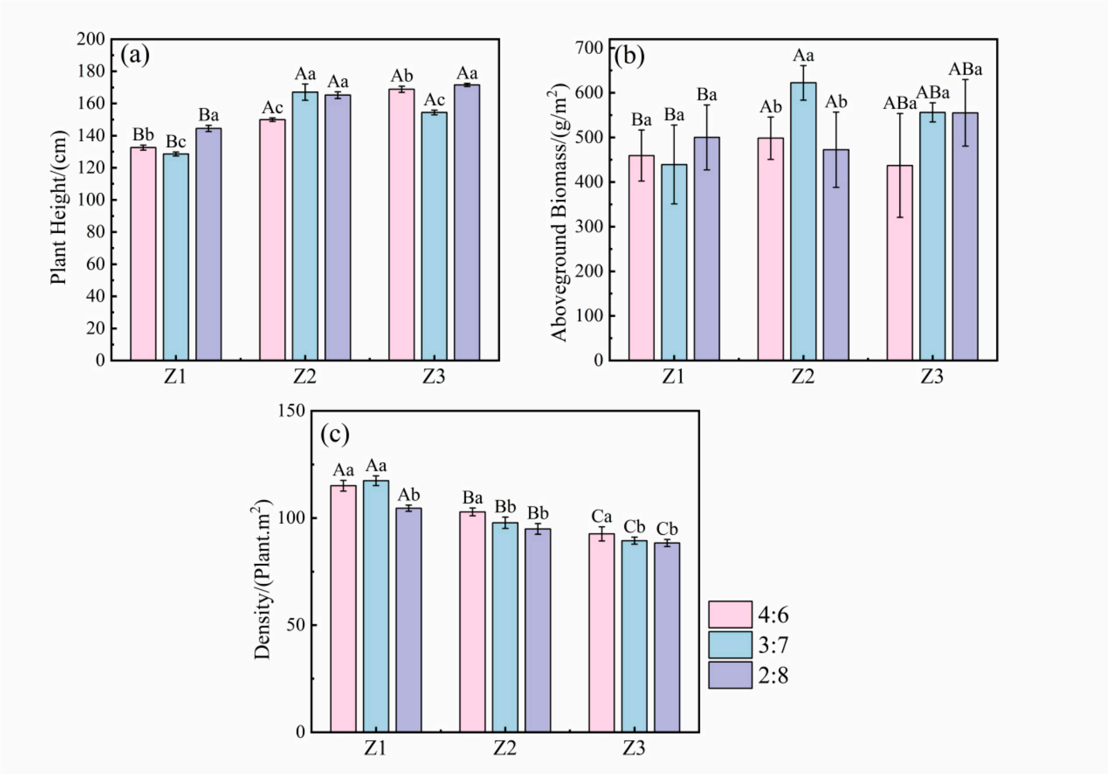

Figure S1. Changes in plant community characteristics under different legume－grass mixture combinations and ratios. (a) Plant height; (b) aboveground biomass; and (c) plant density. Data are presented as mean ± standard error (SE) (n = 3 independent replicates).

Z1, Z2, and Z3 represent three-, four-, and five-species mixtures, respectively. Bars in pink, blue, and purple represent legume-to-grass ratios of 4:6, 3:7, and 2:8, respectively.

Different uppercase letters indicate significant differences among species combinations, whereas different lowercase letters indicate significant differences among mixture ratios within the same species combination ($P < 0.05$).

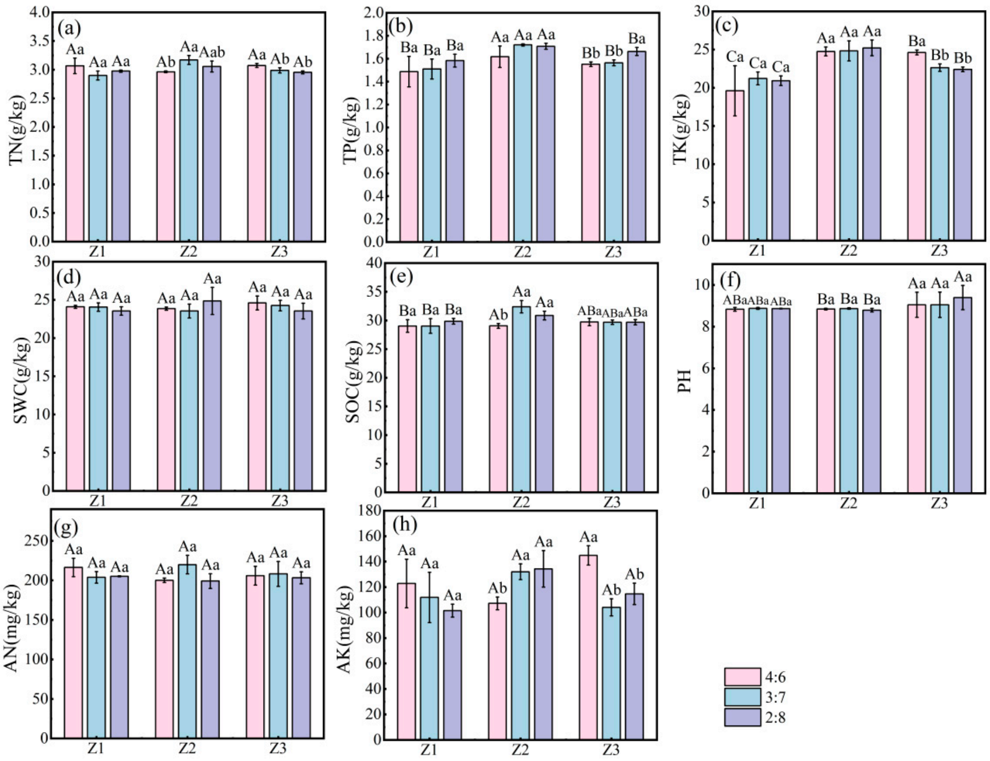

Figure S2. Changes in soil physicochemical properties under different legume–grass mixture combinations and seeding ratios. (**a**) Total nitrogen (TN); (**b**) total phosphorus (TP); (**c**) total potassium (TK); (**d**) soil moisture content; (**e**) soil organic carbon (SOC); (f) soil pH; (**g**) available nitrogen (AN); (**h**) available potassium (AK). Data are presented as mean ± standard error (SE) (n = 3 independent replicates).

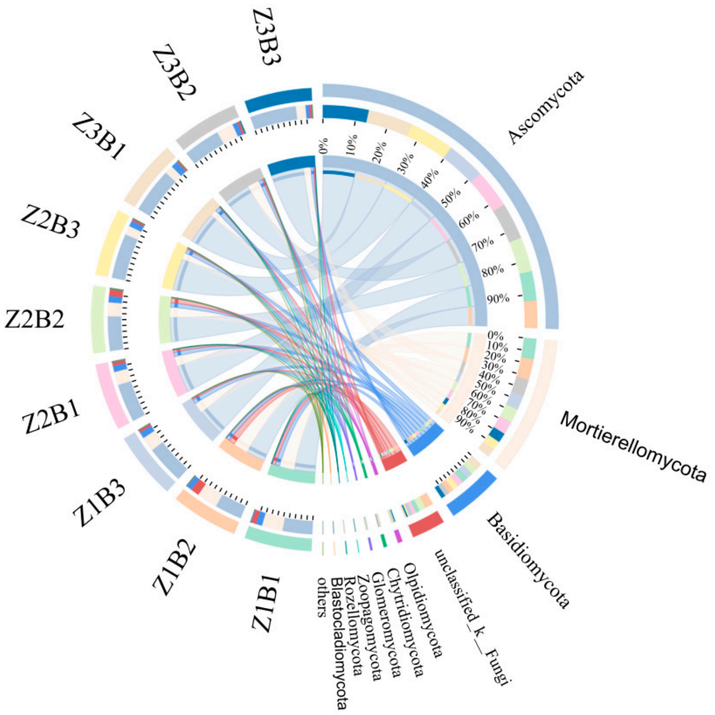

(a)

Figure S3. Chord diagram showing the relationships between fungal phyla and different treatments. The outer circle represents different treatments (Z1B1–Z3B3), while the inner circle represents major fungal phyla. The width of each sector indicates the relative abundance. The connecting ribbons represent the contribution of each fungal phylum to different treatments, with ribbon width proportional to relative abundance.

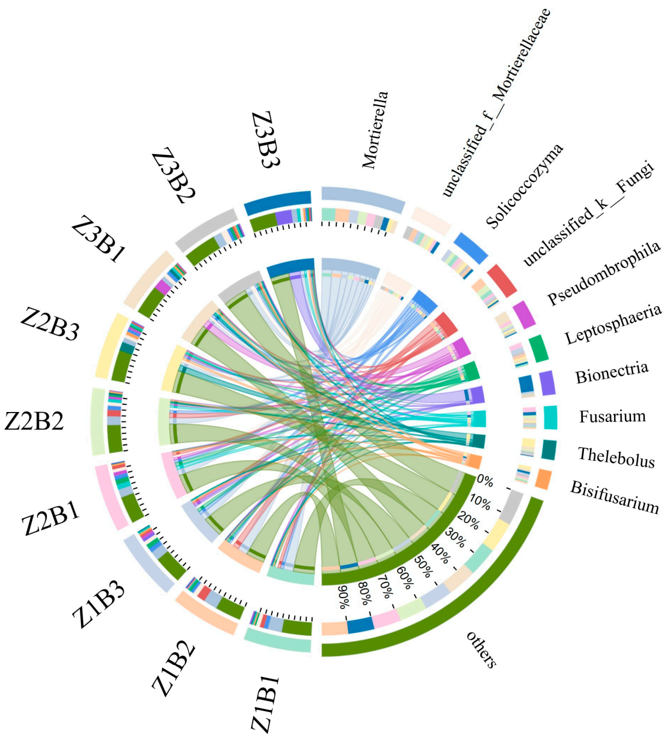

(b)

Figure S4. Chord diagram showing the relationships between dominant fungal genera and different treatments. The outer circle represents different treatments, and the inner circle represents the major fungal genera. The width of each sector indicates relative abundance, and the connecting ribbons illustrate the distribution of each genus across treatments.
