# Supplementary figures and images for: Effects of Legume–Grass Mixture Composition and Seeding Ratio on Plant Community Traits, Soil Physicochemical Properties, and Soil Fungal Diversity
Source: J Fungi (Basel). 2026 May 11;12(5):353. doi: 10.3390/jof12050353 (PMC13208266; doi:10.3390/jof12050353)

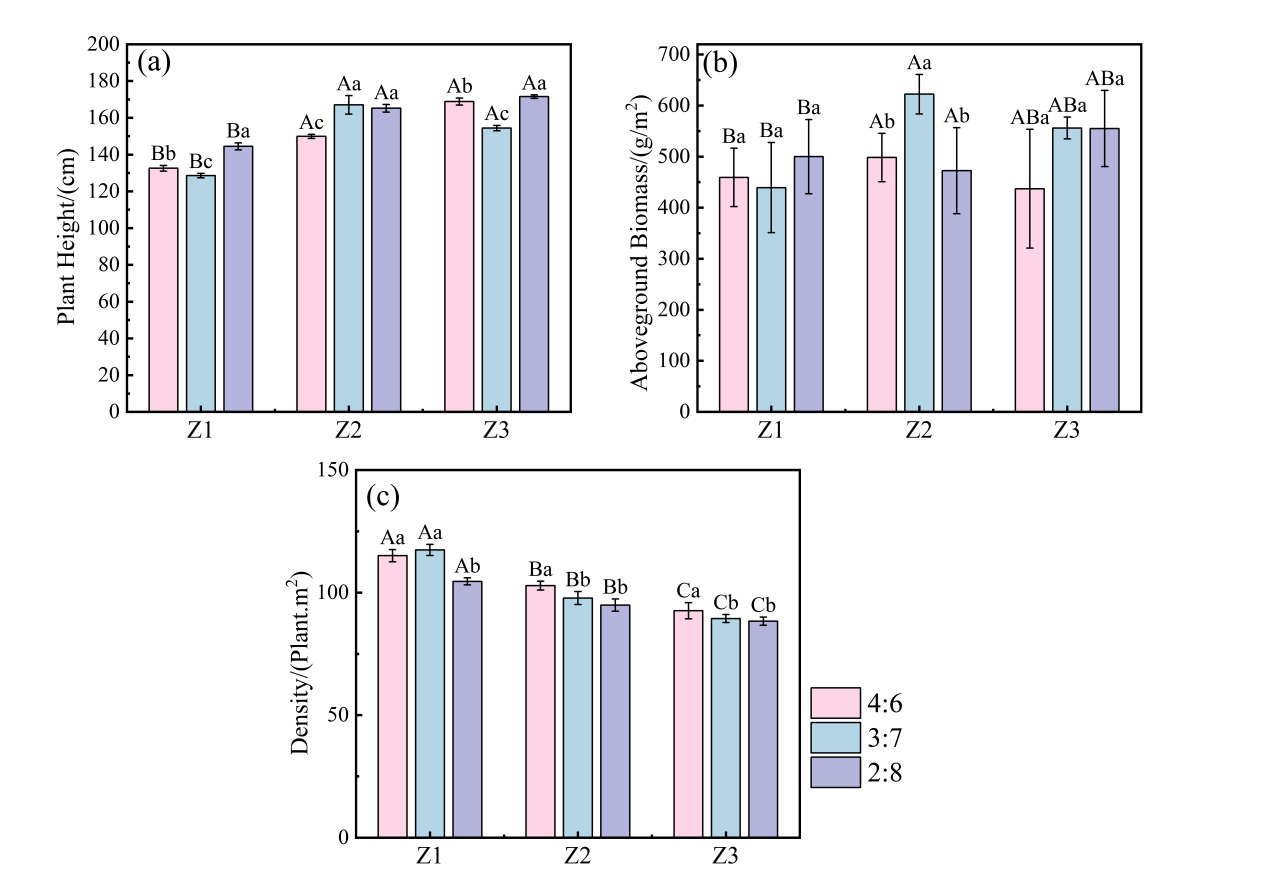

Supplement: Supplementary file 1 [file jof-12-00353-s001.zip › Supplementary_Figure_S1.png]

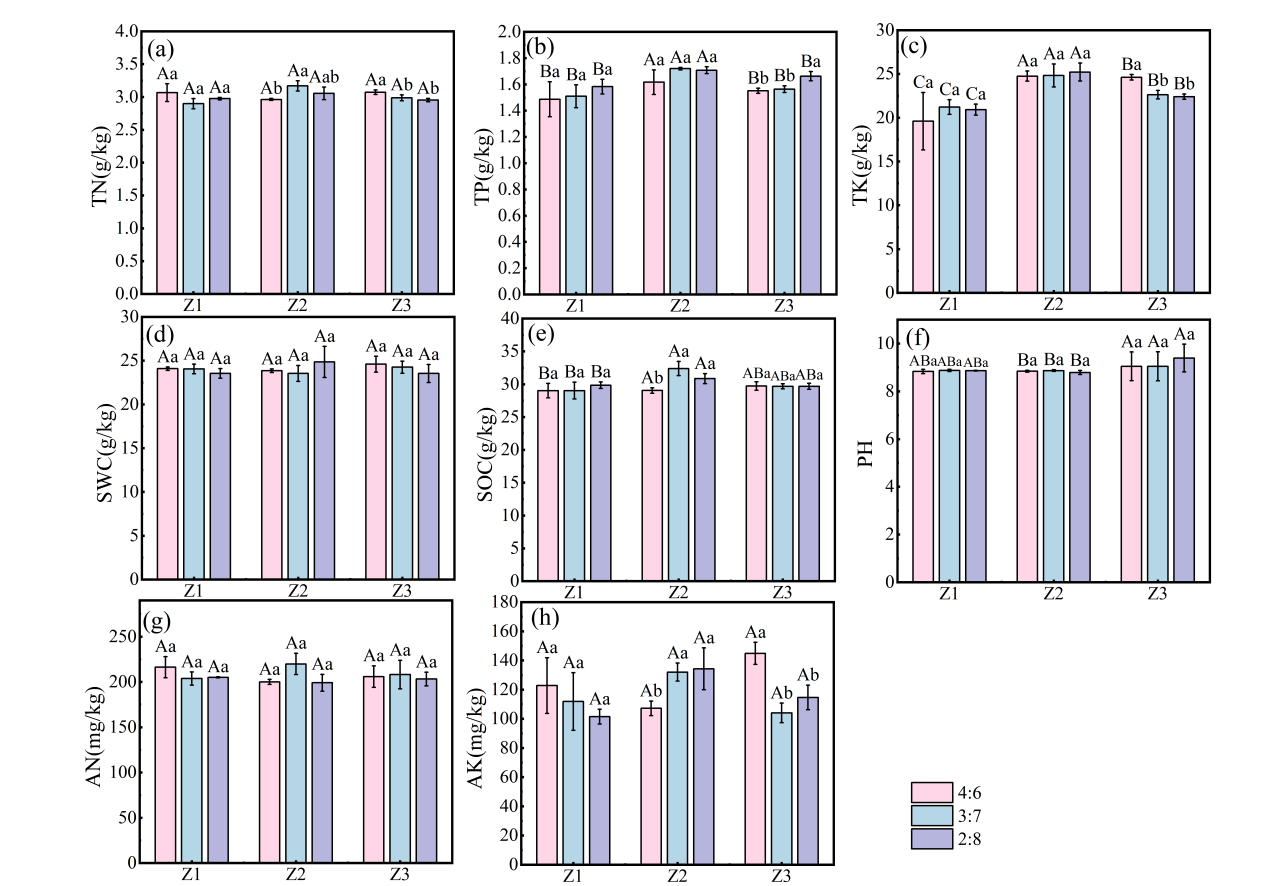

Supplement: Supplementary file 1 [file jof-12-00353-s001.zip › Supplementary_Figure_S2.png]

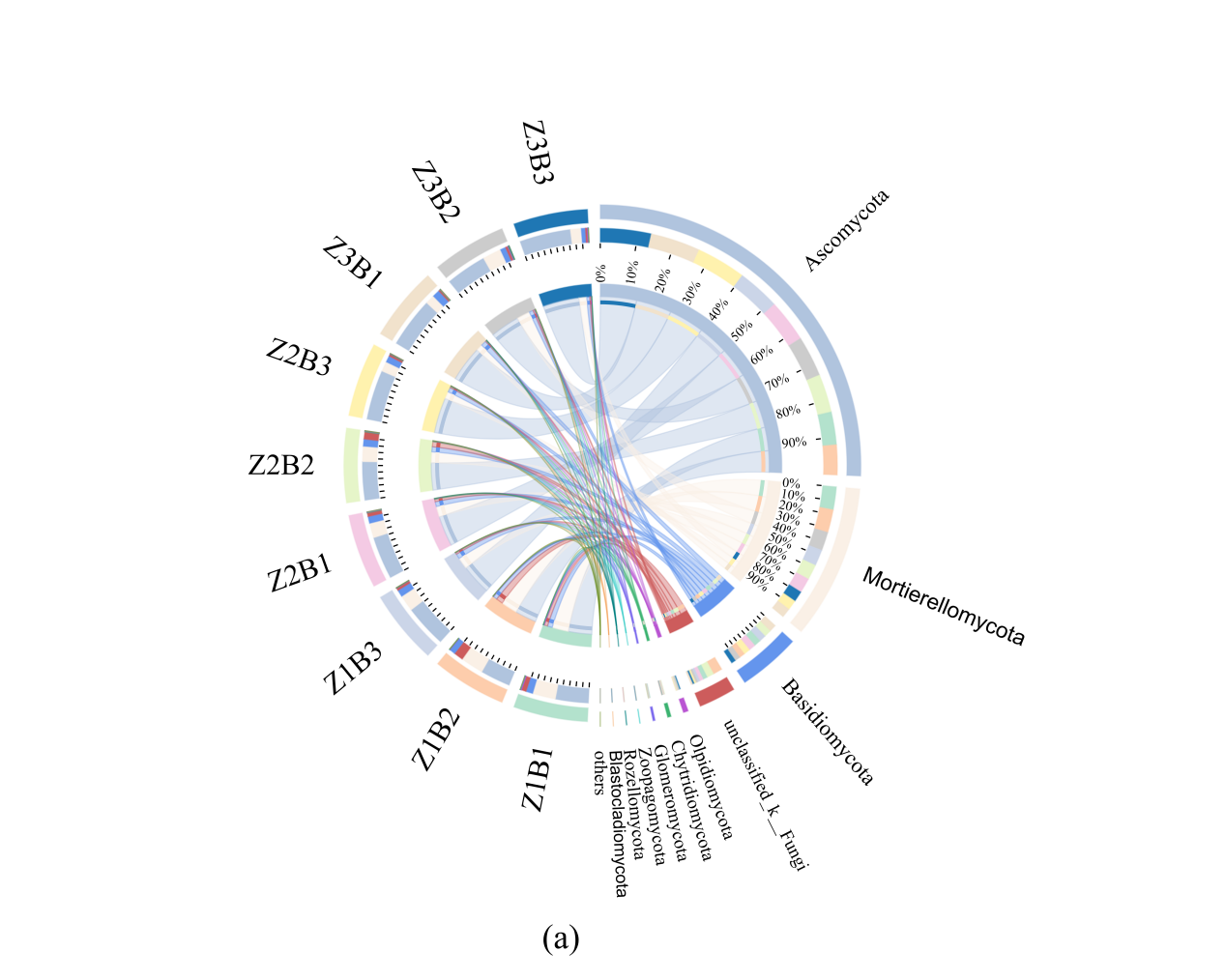

Supplement: Supplementary file 1 [file jof-12-00353-s001.zip › Supplementary_Figure_S3.png]

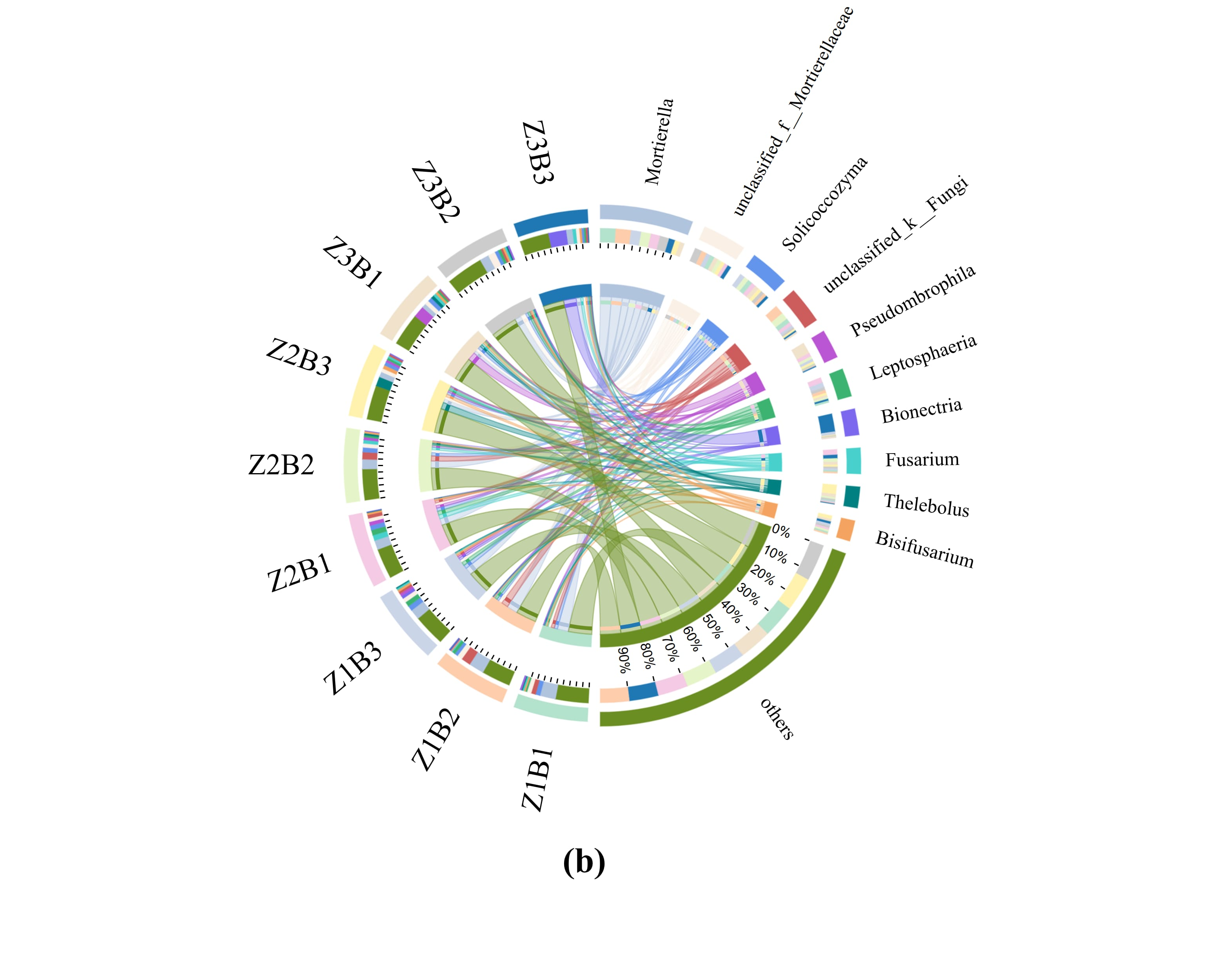

Supplement: Supplementary file 1 [file jof-12-00353-s001.zip › Supplementary_Figure_S4.png]
